# Efficacy and safety of fire acupuncture for psoriasis vulgaris: A protocol of systematic review and meta-analysis of randomized clinical trials

**DOI:** 10.1097/MD.0000000000025038

**Published:** 2021-03-26

**Authors:** Xueli Cheng, Jiawen Lai, Yuelin Zhang, Liyun Lin, Donghan Xu, Zhenghong Zhong, Qibiao Wu, Jing Liu

**Affiliations:** a Macau University of Science and Technology, College of Traditional Chinese Medicine, Macau; b The First Clinical Medical College; c Guangzhou University of Chinese Medicine, Baiyun District, Guangzhou, Guangdong Province, China.

**Keywords:** effectiveness, fire acupuncture, meta-analysis, psoriasis, security, vulgaris

## Abstract

**Introduction:**

Fire acupuncture is commonly used for the treatment of psoriasis vulgaris, but the efficacy and safety of fire acupuncture for psoriasis vulgaris remain unclear.

**Methods:**

This systematic review and meta-analysis will be conducted and reported strictly according to Preferred Reporting Items for Systematic Reviews and Meta-Analyses (PRISMA) guidelines. Five databases including China National Knowledge Infrastructure (CNKI), Wanfang, VIP, Chinese biomedical literature, and Pubmed will be retrieved for potentially eligible studies from their inception to Jan. 2021. All randomized clinical trials comparing fire acupuncture versus no fire acupuncture in the treatment of psoriasis vulgaris will be retrieved and assessed for inclusion. RevMan5.3 software provided by Cochrane collaboration will be used for the analysis. Randomized Clinical Trials Data will be extracted by 2 researchers independently, risk of bias of the meta-analysis will be evaluated based on the Cochrane Handbook for Systematic Reviews of Interventions. The primary endpoint is the total effective rate, the secondary outcomes are the Psoriasis Area Severity Index (PASI) score, the recurrence rate and the adverse reactions.

**Results:**

This study will systematically evaluate the efficacy and safety of fire acupuncture for psoriasis vulgaris. The results will be published in a peer-reviewed journal.

**Conclusion:**

This systematic review will evaluate the effects of fire acupuncture in patients with psoriasis vulgaris, thus providing evidence to the clinical application of this therapy.

## 1 Introduction

Psoriasis, commonly known as psora, is an autologous chronic inflammatory dermatosis in the epidermis and dermis. Its main features consist of thickening of the epidermis and dilation of dermal capillaries, resulting in the aggregation of inflammatory cells in the skin with the formation of erythema and scaly plaques. Other clinical manifestations mainly include pruritus, scales, swelling and pain, etc. Psoriasis usually occurs for the first time between 15 and 25 years old. Arthritic psoriasis often occurs between 30 and 50 years old. However, it can also happen at any age.^[1]^ Combined with the current domestic and foreign studies, the pathogenesis of psoriasis is closely associated with inheritance,^[2]^ infection, immunity, endocrine, environment, mental stress,^[3]^ etc.

Psoriasis treatment options include topical therapy (corticosteroids, vitamin D analogues, retinoids, calcineurin inhibitors, etc), light therapy (phototherapy), and oral or injected medication (steroids, retinoids, methotrexate, cyclosporine, etc), although these therapies help to ease the symptoms of psoriasis, the overall clinical efficacy is not satisfying and some may cause adverse reactions. There is a pressing need for optimal alternative therapy for psoriasis.

Acupuncture is a traditional and complementary method to treat diseases including psoriasis in China and many countries. Fire acupuncture is one kind of acupuncture treatment by inserting a heated needle into an acupuncture point to relieve the symptoms of patients, which has been widely used for the treatment of psoriasis and achieved unique advantages. However, there is a lack of strong evidence for the efficacy of fire needle therapy in the treatment of psoriasis. In this study, we hope to systematically evaluate its safety and effectiveness in order to provide strong evidence for clinical practice.

## 2 Methods

### 2.1 Study registration

The protocol of the systematic review has been registered (OSF Preregisration: https://osf.io/h6j89, January 6, 2021). This systematic review and meta-analysis will be conducted strictly according to Preferred Reporting Items for Systematic Reviews and Meta-Analyses (PRISMA)^[4]^ statement guidelines, and the important protocol amendments will be documented in the full review.

### 2.2 Inclusion and exclusion criteria for study selection

#### 2.2.1 Inclusion criteria

The studies included in this study should meet the following criteria:

(a) All included studies should be randomized controlled trials (RCTs);
(b) The patients in the control group were treated with traditional Chinese medicine, Chinese patent medicine, western medicine, or placebo; those in the treatment group was treated with fire acupuncture besides the treatments used in the control group;
(c) At least one of the following outcome measures was included: total effective rate, the Psoriasis Area Severity Index (PASI) score, recurrence rate, adverse reactions;
(d) Patients did not suffer from other immune diseases, other skin diseases, or cancers;^[5,6]^
(e) Patients did not receive other kinds of acupuncture therapy;
(f) The age or the sex of patients is not limited.

#### 2.2.2 Exclusion criteria

A study should be excluded if it meets one of the following criteria:

(a) Observational study;
(b) Basic study;
(c) Duplicated reports;
(d) Insufficient data^[7]^;
(e) Clinical study with sample size less than 15;
(f) The participants were not psoriasis vulgaris patients;
(g) Due to the different pathogenesis and mechanism, RCTs recruiting patients with erythrodermic psoriasis, pustular psoriasis, arthropathic psoriasis will be excluded.

### 2.3 Types of participants

All included patients were diagnosed with psoriasis vulgaris, regardless of the degree and possible complications. All patients in the treatment group were treated with fire acupuncture.

### 2.4 Interventions

The patients in the control group were treated with placebo or conventional treatments (including traditional Chinese medicine, Chinese patent medicine, western medicine); Those in the treatment group were treated with fire acupuncture besides the conventional treatments. The conventional treatments in both groups are identical, the use of fire acupuncture is the only difference between 2 groups.

### 2.5 Types of outcome measures

The total effective rate was defined as the primary outcome, the PASI score, the recurrence rate, and the adverse reactions were the secondary outcomes.

### 2.6 Search methods

#### 2.6.1 Search resources

**Figure 1:**
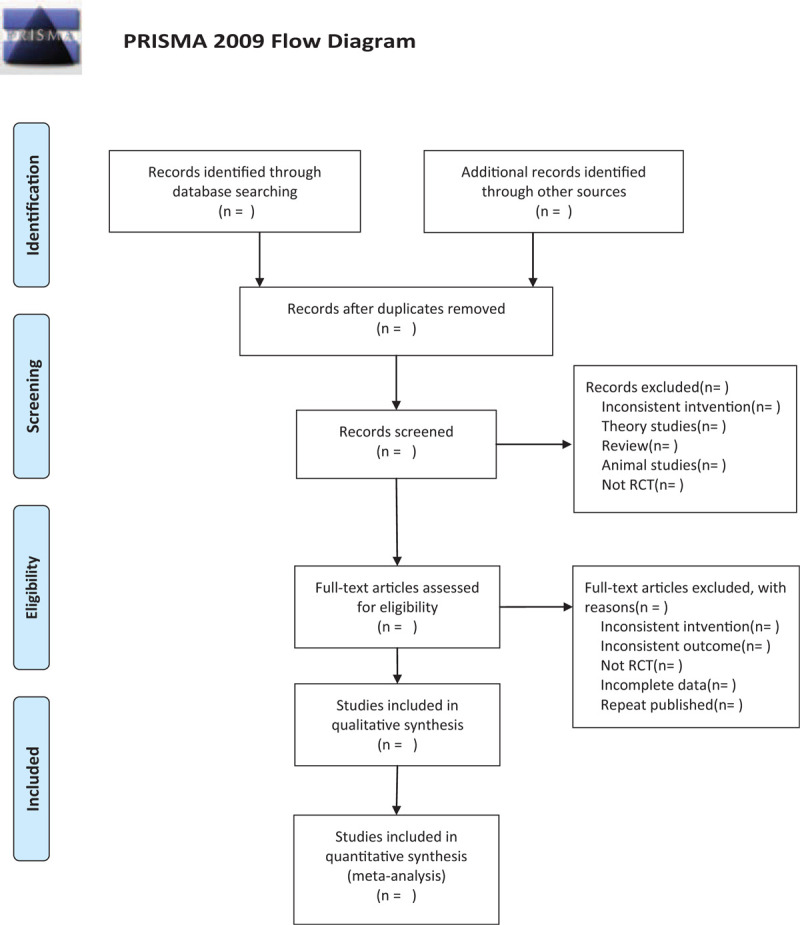
The research flowchart. This figure shows the identification, screening, eligibility, and included when we searching articles.

We will search the following electronic databases from their inception to July 2020: Electronic database includes PubMed, Embase, Cochrane Library, Chinese Biomedical Database WangFang, VIP medicine information, and China National Knowledge Infrastructure (CNKI) (Fig. 1).

#### 2.6.2 Search strategies

The following MeSH terms and their combinations will be used to search:

(1) Fire acupuncture;
(2) Vulgaris;
(3) Psoriasis;
(4) Effectiveness;
(5) Security;
(6) Meta-analysis.

The search strategy for PubMed is shown in Table 1. Other electronic databases will be searched using the same strategy.

**Table 1:** Search strategy in PubMed database.

#1 randomized controlled trial [Title/Abstract]
#2 controlled clinical trial [Title/Abstract]
#3 randomized [Title/Abstract]
#4 randomly [Title/Abstract]
#5 trial [Title/Abstract]
#6 # OR #2 OR #3 OR #4 OR #5
#7 Fire acupuncture [Title/Abstract]
#8 Vulgaris[Title/Abstract]
#9 Psoriasis[Title/Abstract]
#10 #7 OR #8 OR #9
#11 Effectiveness[Title/Abstract]
#12 meta-analysis[Title/Abstract]
#13 Systematic review[Title/Abstract]
#14 #11 OR #12 OR #13
#15 #6 OR #10 OR #14

### 2.7 Data collection and analysis

#### 2.7.1 Studies selection

Two researchers (XC and JL) will independently carry out the selection of studies using Note Express software. Preliminary selection will be performed by screening the titles and abstracts. Then we will download full texts of the relevant studies for further selection according to the inclusion criteria. If there is any different opinion, 2 researchers will discuss and reach an agreement. If a consensus cannot be reached, there will be a third researcher (QW) who makes the final decision. The details of selection process will be displayed in the PRISMA flow chart.

#### 2.7.2 Data extraction

Two researchers (XC and JL) will read the full texts of all included studies, and independently extract the following information:

(a) general information, including trial name and registration information;
(b) trial characteristic, including trial design, location, setting, and inclusion/exclusion criteria;
(c) the characteristics of the participants, including age, race/ethnicity, course of illness, etc;
(d) details of intervention, including acupoints, time of intervention, course of treatment, time of single treatment, etc;
(e) details of comparison interventions;
(f) outcomes as described under Section 2.5.

If we cannot reach an agreement, a third researcher (JL) will make the final decision. One researcher (QW) will contact the corresponding author by telephone or e-mail for more information when the reported data will be insufficient or ambiguous.

#### 2.7.3 Assessment of risk of bias

All the included studies will be evaluated based on the guidelines of Cochrane Handbook for Systematic Reviews of Interventions.^[8]^ The quality of each trial will be categorized into “low,” “unclear,” or “high” risk of bias according to the following items: adequacy of generation of the allocation sequence, allocation concealment, blinding of participants and personal, blinding of outcome assessors, incomplete outcome data, selected reporting the results, and other sources of bias (such as comparable baseline characteristics, inclusion and exclusion criteria).

#### 2.7.4 Data analysis

Review Manager 5.3 (Copenhagen: The Nordic Cochrane Centre, The Cochrane Collaboration, 2014) and Comprehensive Meta-Analysis (CMA) 2.0 will be used to combine trials. Continuous outcomes will be expressed as weighted mean difference (WMD) and dichotomous data as risk ratio (RR), with their 95% confidence intervals (CIs). RR is the ratio of the probability of an event occurring in the treatment group to the probability of the event occurring in a control group.^[9–11]^

Besides RR, meta-analysis will be also conducted using odds ratio (OR) and risk difference (RD) statistical methods, respectively. Chi^2^ test and *I*^2^ statistic will be used to measure statistical heterogeneity. When *P* < .1 or *I*^2^ > 50%, substantial heterogeneity will be considered to exist, and the random-effects model will be applied to estimate the summary RR (or OR, RD), WMD and 95% CI, otherwise a fixed-effects model will be applied.^[12–14]^

Sensitivity analysis will be adopted to determine the robustness of the results.

#### 2.7.5 Patients and public involvement

This is a meta-analysis study based on previously published data, so patient and public involvement will not be included in this study.

#### 2.7.6 Ethics and dissemination

This study is a systematic review, the outcomes are based on the published evidence, so examination and agreement by the ethics committee are not required in this study. We intend to publish the study results in a journal or conference presentations.

#### 2.7.7 Evidence assessed

The quality of evidence for this study will be assessed by “Grades of Recommendations Assessment, Development and Evaluation (GRADE)” standard established by the World Health Organization and international organizations.^[15]^ To achieve transparency and simplification, the quality of evidence is divided into 4 levels in the GRADE system: high, medium, low, and very low. We will employ GRADE profiler 3.2 for analysis.^[16]^

## 3 Discussion

Psoriasis is a recurrent chronic inflammatory dermatosis with a genetic tendency. It is intractable, generalized, and prone to recurrence. The incidence is related to heredity, streptococcus infection, autoimmune abnormality, etc.

At present, the pathogenesis of psoriasis remains unclear. The glucocorticoid for external use is the main treatment. The long-term use of glucocorticoid may lead to the impairment of skin cuticle, skin barrier function damage, and recurrent attacks after drug withdrawal. It is difficult and refractory to treat psoriasis.

In recent years, TCM dermatologists are gradually getting rich experience in the treatment of psoriasis. The treatments are not limited to the oral administration of TCM decoction, there are a lot of effective methods for the management of this disease. Among them, the treatment methods of psoriasis in TCM include acupuncture, cupping, fire needle, traditional Chinese medicine bath, fumigation, sealing, cupping, bloodletting, acupoint injection, etc. Fire needle is one of the special external therapy of TCM, which is derived from the Large Needle in the ancient “Nine Needles.” Fire needle is also known as a “burning needle” or “red-hot needle.” The needle tip is quickly piercing into the acupoint after burning it red to treat diseases. It has strong effects of strengthening body resistance and eliminating evil, removing necrotic tissue and promoting granulation, dispersing cold and dehumidifying at the targeted lesions via supporting yang, and supplementing fire to destroy and discharge pathogens.^[17]^ It can achieve the effect of “opening the demon door and cleansing the house” through the fire needle. Meanwhile, it helps the blood circulation via warming and dredging meridians, thereby making the heat-toxicity and stasis some way out. Moreover, modern medical research showed that^[18]^ the treatment of psoriasis vulgaris with fire needle can reduce local inflammatory edema, improve the inflammatory infiltration, as well as can improve the phagocytosis of white blood cells and promote the localization of inflammation. Zhang Yan^[19]^ believes that the fire needle can effectively regulate immunity, enhance the detoxification of lymphocytes and phagocytosis of cells, and regulate keratinocytes. Some studies show that adding drug treatment on the basis of fire needle treatment can further improve the therapeutic effect.^[20]^

This study will systematically evaluate the efficacy and safety of fire acupuncture for psoriasis vulgaris, thus providing valuable evidence to this therapy for the patients with psoriasis vulgaris.^[21–23]^

## Author contributions

**Conceptualization:** Xueli Cheng, Jing Liu, Qibiao Wu.

**Data curation:** Jing Liu, Jiawen Lai.

**Formal analysis:** Xueli Cheng, Jiawen Lai.

**Methodology:** Xueli Cheng, Liyun Lin, Zhenghong Zhong.

**Project administration:** Qibiao Wu.

**Resources:** Xueli Cheng, Yuelin Zhang, Donghan Xu.

**Software:** Xueli Cheng, Donghan Xu.

**Supervision:** Qibiao Wu.

**Writing – original draft:** Xueli Cheng.
